# Assessment of Perceptions and Cancer Risks of Workers at a Polychlorinated Biphenyl-Contaminated Hotspot in Ethiopia

**DOI:** 10.5696/2156-9614-11.30.210609

**Published:** 2021-05-28

**Authors:** Sisay Abebe Debela, Ishmail Sheriff, Endashaw Abebe Debela, Musa Titus Sesay, Alemu Tolcha, Michaela Sia Tengbe

**Affiliations:** 1 Department of Public Health, College of Health Science, Selale University, Fiche, Ethiopia; 2 Department of Public Health, College of Human Resource Development, 8 Black Street Drive, off Alusine Kala Drive, Magbenteh, Makeni; Sierra Leone; 3 Department of Internal Medicine, School of Medicine, Adama Hospital Medical College, Adama, Ethiopia; 4 Department of Environmental Engineering, School of Environmental Science and Engineering, Suzhou University of Science and Technology, Suzhou, Jiangsu Province; People's Republic of China; 5 Department of Environmental Health, College of Medicine and Health Science, Hawassa University, Hawassa Ethiopia; 6 Department of Environmental Science and Engineering, School of Environment and Safety Engineering, Jiangsu University, Zhenjiang City, Jiangsu Province; People's Republic of China

**Keywords:** cancer risk, human health, polychlorinated biphenyls, public perception, PCB

## Abstract

**Background.:**

Polychlorinated biphenyls (PCBs) are synthetic and persistent toxic chemicals with a high potential to bioaccumulate in human tissue. There is no existing literature on workers' perceptions and occupational cancer risk due to exposure to PCBs in Ethiopia.

**Objectives.:**

The aim of the present study was to assess workers' perceptions of occupational health and safety measures of PCB management and to evaluate the cancer risk posed by PCBs to workers handling these chemicals in Ethiopia.

**Methods.:**

A total of 264 questionnaires were administered to workers at the study area to obtain information about PCB management. A mathematical model adopted from the United States Environmental Protection Agency (USEPA) was used to assess the potential cancer risk of people working in PCB-contaminated areas.

**Results.:**

The results showed that the majority of the workers had little knowledge of safe PCB management practices. Furthermore, 82.6% had not received training on chemical management and occupational health and safety protocols. The association between respondents' responses on the impact of PCBs to the use of personal protective equipment was statistically significant (p <0.005). Accidental ingestion, dermal contact and inhalation exposure pathways were considered in assessing the cancer risk of people working in these areas. The estimated cancer risk for PCBs via dermal contact was higher than for the accidental ingestion and inhalation pathways. The health risk associated with dermal contact was 73.8-times higher than the inhalation exposure route. Workers at the oil tanker and oil barrel area and swampy site are at higher risk of cancer via dermal contact at the 95th centile (879 and 2316 workers per million due to PCB exposure, respectively). However, there is very low cancer risk at the staff residence and garden area via the inhalation route.

**Conclusions.:**

Training programs would help improve the knowledge of workers in the area of occupational health and safety of chemical handling. Further studies on PCBs in the exposed workers will provide information on their blood sera PCB levels and consequently identify potential health impacts.

**Participant Consent.:**

Obtained

**Ethics Approval.:**

Ethics approval was obtained from the Research Ethics Review Committee of Adama Hospital Medical College, Adama, Ethiopia.

**Competing Interests.:**

The authors declare no competing financial interests.

## Introduction

Polychlorinated biphenyls (PCBs) are chlorinated aromatic hydrocarbons that have a distinct characteristic of a biphenyl ring with an attachment of 1 to 10 chlorine atoms with the chemical formula (C_12_H_10-_*_n_*Cl*_n_* (n=1–10)).^1^,^2^

In theory, there are a total of 209 isoforms or congeners of PCBs.^1^,^3^ The nomenclature of these PCB congeners have been well summarized in the review published by Mills III *et al.*^4^ Polychlorinated biphenyls can be separated into coplanar and nonplanar congeners, depending on the positioning of the two benzene rings.^2^ The literature on PCBs has been well documented. There are a total of 12 types of coplanar PCBs, and their structures are comparable in certain respects to dioxins considering the rotation of phenyl-phenyl groups.^1^ Of these 12 coplanar PCBs, there are eight congeners having only ortho substitution [mono-ortho] in one site [PCBs 105, 114, 118, 123, 156, 157, 167 and 189], while the remaining 4 congeners have no chlorine substituted in the ortho location (PCBs 77, 81, 126 and 169). The toxicological effect of these 12 PCB congeners is similar to those of other pollutants such as polychlorinated dibenzodioxins (PCDDs) and polychlorinated dibenzofurans (PCDFs).^5^ Because of their toxicological similarities with PCDDs and PCDFs, they are referred to as dioxin-like (DL)-PCBs and previous work has documented the differences between dioxin-like PCBs and the other PCBs congeners.^6^ In addition, a meta-analysis study thoroughly detailed the effects of coplanar PCBs on humans.^1^ In contrast, non-coplanar PCBs have chlorine atoms attached to them in the *ortho*-position.^5^ There are 197 non-coplanar PCBs and those congeners are normally referred to as non-DL congeners. The effects of non-planar congeners have also been widely studied.^9^,^10^ Dioxin and non-dioxin PCBs congeners are classified based on toxicological effects endpoints (e.g., endocrine active PCBs, immune-toxic PCBs, neurotoxic PCBs) in addition to different mechanisms of action.

Polychlorinated biphenyls were first synthesized around the early 1880s,^9^ and mass production for commercial uses commenced in 1929.^10^,^11^ The manufacture, use, spill, and improper disposal of PCBs has led to substantial environmental contamination and health hazards. The environmental impacts of PCBs were first reported in 1966.^12^,^13^ Subsequently, PCBs gained global recognition when a PCB mixture, Kanechlor-400, contaminated rice oil, causing Yusho disease in more than 1600 victims in Fukuoka and Nagasaki prefectures in Japan in 1968.^14^,^15^ Clinical features of Yusho disease include headache, grayish dark brown pigmented skin at birth, and general fatigue. Similarly, PCB mixtures Kanechlor 400 and 500 contaminated a rice bran cooking oil (C-rice bran oil) that later caused Yu-Cheng disease in more than 2000 people in Changhua, Taichung, Hsinchu and Miaoli counties in Taiwan in 1979.^16^,^17^ Yu-Cheng disease is characterized by pigmentation of the skin and nails, acne, and hypersecretion of the meibomian glands. Human exposure to PCBs is mostly through consumption of PCB-contaminated food. For instance, PCB concentrations ranged between 41.8–77.7 ng g−1 (lipid weight) in a study of meats (beef, pork, chicken and turkey) from Italy.^18^ Furthermore, PCBs were detected in the muscle of edible fish species such as sardine (4.15–17.9, ng/g w.w.), anchovy (1.01–7.08 ng/g w.w.) and bogue (1.46–7.22 ng/g w.w.) in the Mediterranean Sea.^19^ The United States Environmental Protection Agency (USEPA) toxicological reference values for PCBs in food, water and air are 3 ppm, 5E-4 and 1 mg/m^3^ respectively.^20^ Humans can also be exposed to PCBs via occupational practices.^21^–^23^ In 1978, 12 000 people were occupationally exposed to PCBs in the United States.^24^ Similarly, the mean concentration of blood PCB levels of female workers that had been handling Kanechlor 300 in Japan was found to be 32.3 ± 20.6 ppb, 10 to 100 times higher than that of non-occupationally exposed mothers.^25^ Occupational mortality studies of PCB-exposed workers or groups have been well summarized.^2^, ^26^

**Abbreviations**
*DL*: Dioxin-like
*PCB*: Polychlorinated biphenyl
*USEPA*: United States Environmental Protection Agency

Polychlorinated biphenyls cause different toxicological effects on humans depending on the position of chlorine substitution on the rings. For example, it was reported that changes in levels of thyroid hormone among Vietnamese e-waste workers was due to PCB concentration in serum.^27^ A cohort study in northern Italy reported a positive correlation between total concentration of PCBs in serum and the onset of dementia.^28^ A study by Wang *et al.* found that exposure of e-waste workers to PCBs resulted in DNA damage in the lymphocytes as well as in spermatozoa.^29^ A recent systematic review and meta-analysis revealed that PCB exposure, particularly to DL-PCBs, may be a risk factor for hypertension.^30^ Cancer is a leading cause of mortality globally and it has been linked to toxic chemicals. Polychlorinated biphenyls are classified as Group 1 carcinogens by the International Agency for Research on Cancer (IARC).^24^ A recent study found that PCB74, PCB99, and PCB118 were associated with 5-year, but not longer-term, breast cancer-specific mortality in women in North Carolina.^31^ Some systematic review and meta-analysis studies have detailed the association between PCB exposure and risk of some cancers. 32–34

Polychlorinated biphenyls were among the first compounds designated as persistent organic pollutants (POPs) on 22 May 2001 by the Stockholm Convention.^35^,^36^ The convention entered into force on 17 May 2004 with 128 parties and 151 signatories initially and in 2019 there are 184 parties on board.^37^ However, there remains a high accumulation of PCB-containing materials around the globe. Polychlorinated biphenyls are still in active use in equipment such as capacitors and transformers.^38^ For example, in Gambia, a total of 19 PCB-containing transformers were in active use in 2015.^36^ In Myanmar, a recent assessment found that 119 transformers exceeded the 50 ppm threshold of the Stockholm Convention chlorine content. Of these, 110 transformers containing PCBs are in active use.^39^ Current estimates suggest that about 17% of PCBs globally have been eliminated, with 83% remaining.^40^,^41^ In Ethiopia, PCBs have been imported into the country in electronic materials and transformers. Ethiopia's national inventory on POPs indicated the presence of a huge stockpile of PCB-containing equipment across the country. Furthermore, the improper disposal and dumping of transformers and capacitors has resulted in serious leakage of PCB oil.^42^ This constitutes a health risk as e-waste processing is a major source of human exposure to hazardous environmental pollutants.^43^ A study by Debela *et al.*^44^ found high levels of PCBs in soil from a hotspot in Ethiopia. It is therefore necessary to assess the potential cancer risk of workers who work at these sites who are exposed to PCBs daily.

The United Nations Sustainable Development Goal (SDG) 3.9 emphasizes the need to substantially reduce the mortality and morbidity due to hazardous chemicals.^45^ To the best of our knowledge, there are no studies on the human health risk associated with exposure to chemicals, oil, and equipment containing PCBs in Ethiopia, although Diribe and colleagues conducted a human health risk assessment using PCB levels found in fish muscle tissues.^46^ In order to address this knowledge gap, the aim of the present study was to examine the health risks associated with exposure to PCBs. The objectives were to examine workers' perceptions of the health risk of PCBs and evaluate cancer risk due to PCBs via different exposure scenarios.

## Methods

**Figure 1:**
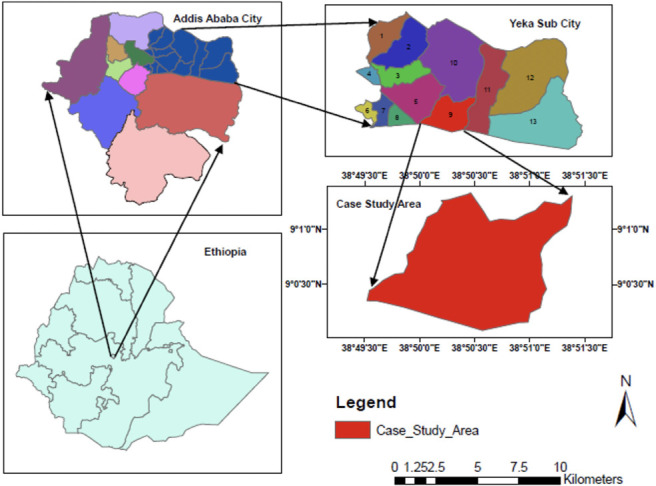
Study area in Addis Ababa, Ethiopia^44^

**Figure 2:**
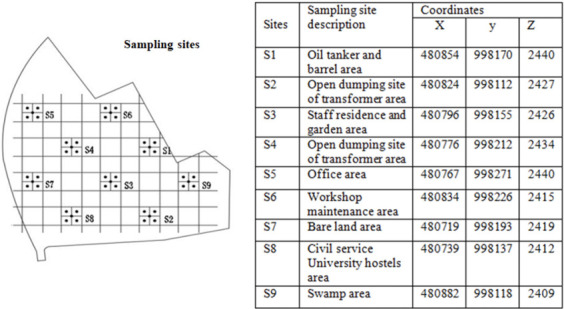
Map of nine study area sampling sites in Addis Ababa, Ethiopia^44^

The present study was carried out in Addis Ababa, the capital city of Ethiopia, which serves as the headquarters of the African Union (AU). The geographical features of the city have previously been described.^47^ Details of the demography and site-specific information of the study sites have recently been reported in our previous publication.^44^ In brief, the study area is the Kotobe workshop situated in Yeka sub city, Addis Ababa. It is used for repair and maintenance of transformers and capacitors. The area is being used as a dumpsite for equipment consisting of PCBs that are beyond repair or at the end of their useful life span. Due to the nature of the activities in the area, it is being recognized as a national PCB-contaminated site (*Figure 1*). The study area has nine sampling sites and each of these showed different activities with regard to PCB management (*Figure 2*). For example, in site 1 (S1), there is a large accumulation of tankers and barrels with PCB-containing oil.

**Figure 3:**
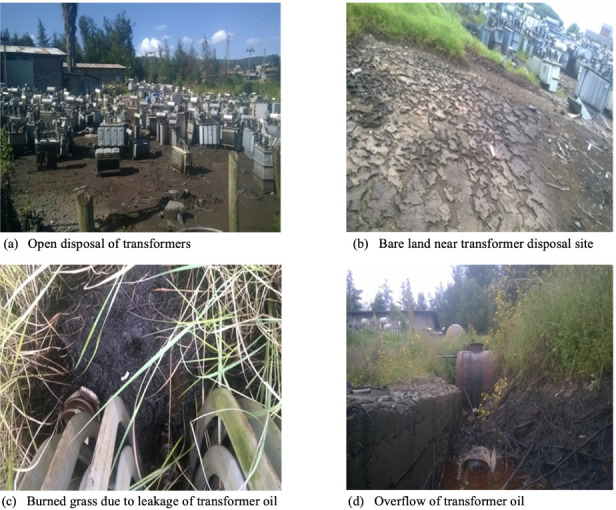
Existing management of PCB-containing transformers and other equipment^44^

### Research design

For the questionnaire survey, a cross-sectional study was conducted over the course of three months (June to August 2020). Taking into account the inherent complexity of the study objectives and the background of the study participants, two hundred and sixty-four (264) workers were selected for the questionnaire interview on PCBs. The participants were workers whose routine activities include the handling of transformer oil, discarded PCB-related materials as well as the maintenance of PCB-containing equipment and who consented to participate in the study.

The questionnaires were distributed to the various departments at the site using the workforce/staff statistics obtained from the human resources department. Study participants were selected from each department/division using a systematic random sampling technique based on their willingness to participate in the interview. The sample size was calculated using Equation 1 as reported by Glenn.^48^

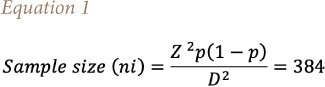
Where: z =1.96 for the 95% confidence level, p = percentage selecting a choice (50%), with a confidence interval of 95% or 5% of margin of error (D). The source population of the study area is less than 1000 people (638), so the corrected infinite sample size (nf) was calculated using Equation 2, where N = population size.

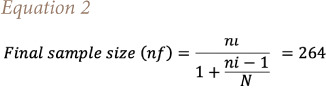



### Data collection

Data collection was conducted through face-to-face interview with the respondents. The interviewers were recruited from the Ethiopian Environment and Forest Research Institute based on academic qualification and familiarity with the topic. They were trained over three days on the content of the questionnaire and on interview techniques. Before starting field visits, the questionnaire format was tested to ensure the questions were clear and to estimate the duration of questionnaire administration. This field validation led to slight modification of the questionnaire. The questionnaire can be found in Supplemental Material 1.

Informed consent was sought from each participant by providing them with an information sheet stating the objectives of the research and informing them that their participation was voluntary. The issue of confidentiality and anonymity of participants' responses was also highlighted. The content of the information sheet was translated into the local language best understood by each participant, although most of them had good level of English proficiency. It was made clear to participants that they could refuse any questions posed to them during the interview session.

The interview questions were centered on different themes such as exposure duration (ED), as well as exposure frequency (EF) of workers to PCBs and PCB-containing materials, their impact on human health and the environment, training on hazardous chemical management, and protective measures for the use and maintenance of PCBs and related materials. In brief, data were collected on level of awareness, knowledge, attitudes, and practice of handling and disposing of PCBs, and environmental and health risks associated with PCBs in the study area.

Ethical approval was obtained from the research ethics review committee of Adama Hospital Medical College, and consent to participate was guaranteed by the ethics review committee. All participants were informed about the study and participation was voluntary. Confidentiality and privacy of the data were considered.

#### Health risk characterization and exposure scenarios

Polychlorinated biphenyl concentration data obtained in our previous study were used to calculate cancer risk.^44^ The site characteristics are presented in Figure 2. The referenced study used both quantitative and qualitative methods of data collection.^44^ For the quantitative method, forty-five (45) soil samples were collected from the nine sampling sites and tested at a laboratory for PCB content. In the qualitative approach, a checklist was used to assess the sites condition and management of PCB-containing equipment and oil.^44^

In the current study, accidental soil ingestion, dermal contact (dermal absorption of pollutant) and inhalation of fugitive particles of contaminated soil were considered as exposure pathways for estimating lifetime intake. In Ethiopia, there is no officially recommended guideline for cancer risk assessment, therefore calculations were adapted from the USEPA. The potential cancer risk of workers and residents who are in daily contact with PCB-related materials was estimated based on Equation 3,^49^ 4,^50^ and 5.^51^


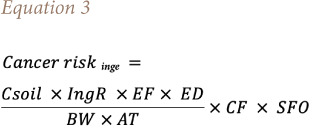


Where Cancer risk_inge_ = cancer risk by accidental soil ingestion, C_soil_ = concentration of the PCB in soil (mg/kg), IngR = ingestion rate of soil (mg/day), EF = exposure frequency (days/year), ED = exposure duration (years); BW = average body weight (kg); AT = averaging time (days); CF = conversion factor (1 × 10^−6^ kg/mg); and SFO = oral slope factor (mg/kg/day) ^−1^.


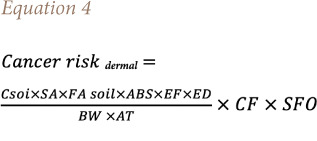


Where Cancer risk _dermal_ = cancer risk by dermal contact of soil, SA = surface area of workers' skin that comes into contact with soil (cm^2^/day), AF_soil_ = adherence factor of skin for soil (mg/cm^2^), and ABS is the dermal absorption factor.


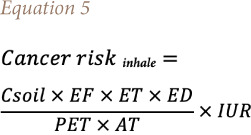


Where Cancer risk _inhale_ = cancer risk due to inhalation of soil particles, ET = exposure time (h/day), IUR = inhalation unit risk (mg/m^3^) ^−1^, AT = averaging time (h), and PEF is the particle emission factor = 1.36 × 10^9^ m^3^/kg. Particle emission factor is the inhalation of pollutants adsorbed to respirable matter via fugitive dust emissions from contaminated soils which relates to the concentration of a pollutant in soil and respirable particles (PM_10_) in the air.^52^ The USEPA defines the IUR as the lifetime cancer risk estimated to result from prolong exposure to a substance at a level of 1 μg/m^3^ in air.^51^

The variables listed in Tables 2 and 3 are used for human cancer risk estimation. The result of calculated lifetime cancer risk is described as: very low ( 10^−6^), low (10^−6^ <to<10^−4^) , moderate (10^−4^ < to < 10^−3^) , high (10^−3^ to < 10^−1^) and very high (10^−1^).^53^ Oral slope factor (mg/kg/day)^−1^, SFO (mg/kg-day)^−1^ and IUR (mg/m3)^−1^ of BaP are listed in Table 2.^54^ Slope factor is plausible upper-bound estimate of the probability of a response per unit intake of a chemical over a lifetime. The slope factor is used to estimate an upper-bound probability of an individual developing cancer as a result of reference values. 50

### Data analysis and quality control

The interviewers checked the administered questionnaires on a daily basis to ensure the information captured was complete and to identify possible errors before data entry commenced. The personal data of all study participants recorded on the administered questionnaire were de-identified to ensure confidentiality. Statistical analysis of the data was done using the Statistical Package for the Social Sciences (SPSS) version 23.0 to determine the distribution and association between variables using the chi-square test and cross tabulation. The mathematical model used to calculate the cancer risk was adopted from the USEPA, and Microsoft Excel software was used to perform the calculations. Differences in proportions were computed to determine significance using the chi-square test and a p-value < 0.05 was taken as statistical significance. An interpretive approach was used for analysis of the questionnaire data focusing on the respondents' perceptions and interpretation of the topics discussed in the interview sessions. Questionnaires were not analyzed to show whether the perception of a participant or a group of participants on a particular theme was wrong or correct, as perception can be influenced by different factors including education level. Rather, the analysis focused on the thoughts of the participants on the topics captured in the questionnaire and related them to the existing knowledge available in the wider literature.

### Polychlorinated biphenyl-related data

Potential health risk exposure of an adult worker to DL-PCBs and non-DL-PCBs via accidental ingestion of soil particles, dermal contact pollutant absorption, and inhalation of fugitive soil particles was estimated using the concentration data in Table 1. Table 1 shows the distribution of PCBs in soil from the Kotobe warehouse maintenance workshop and transformer dumping site in Addis Ababa (study area) of 18 congener PCBs.

**Table 1:** Polychlorinated Biphenyl Concentration Congeners Across Study Sites (mg kg^−1^ dw)^44^

	**S1**	**S2**	**S3**	**S4**	**S5**	**S6**	**S7**	**S8**	**S9**
**PCB28**	2.15	0.214	0.23	0.0721	0.195	0.473	0.459	0.0445	0.505
**PCB51**	1.71	0.23	0.153	0.104	0.151	0.171	0.442	0.116	0.177
**PCB101**	0.0785	0.0195	0.0139	0.0671	0.00302	0.0202	0.0077	0.00601	0.0042
**PCB81**	0.175	0.21	0.147	0.19	0.127	0.214	0.0988	0.0813	0.173
**PCB77**	0.132	0.183	0.149	0.103	0.08	0.114	0.0691	0.0729	0.183
**PCB123**	0.0152	0.0172	0.0339	0.303	0.0252	0.0838	0.0512	0.0246	0.0217
**PCB118**	0.0262	4.38E-3	0.0124	0.184	0.00751	ND	0.0251	0.0259	0.00634
**PCB114**	0.0358	0.0228	0.0168	0.202	0.0109	0.0627	0.0168	0.0122	0.0247
**PCB153**	0.0662	0.0491	0.0113	0.0756	0.0445	0.044	0.0268	0.0351	0.0398
**PCB105**	ND	0.0162	0.0293	0.185	0.0125	0.265	0.0665	0.00253	0.0235
**PCB138**	0.0828	0.0576	0.00498	0.152	0.0323	0.0949	0.0449	0.0294	0.0158
**PCB126**	ND	0.0259	0.0759	0.123	0.0416	0.0404	0.0221	0.0294	0.0822
**PCB167**	0.038	0.0279	0.0122	0.133	0.00655	0.0719	0.0631	0.13	0.0653
**PCB156**	0.0407	0.0201	0.0275	0.038	0.00872	0.0556	0.0774	0.0774	0.146
**PCB157**	0.0462	0.0122	0.00186	0.033	0.0534	0.078	0.0247	0.147	0.187
**PCB180**	ND	ND	0.00438	0.0238	0.0404	0.0439	0.0234	0.147	0.187
**PCB169**	0.0944	0.0518	0.0757	0.0888	0.0189	0.0737	0.0972	0.0896	0.256
**PCB189**	0.171	0.163	0.137	0.0208	0.169	0.00191	0.00422	0.00593	0.018

ND: Non-detected PCBs

**Table 2:** Slope Factor of Polychlorinated Biphenyl Congeners for Ingestion (SFO (mg/kg-day)^−1^, Dermal Contact (SFO × GIABS (mg/kg-day)^−1^), Inhalation (mg/m^3^)^−1^) and Dermal Absorption Factors^54^

**PCB**	**SFO (mg/kg-day)^−1^**	**IUR (Ug/m^3^)^−1^**	**RFDo (mg/kg-day)**	**RFCi mg/m^3^**	**ABS**
189	3.9E+00	1.1E-03	2.3E-05	1.3E-03	0.14
167	3.9E+00	1.1E-03	2.3E-05	1.3E-03	0.14
157	3.9E+00	1.1E-03	2.3E-05	1.3E-03	0.14
156	3.9E+00	1.1E-03	2.3E-05	1.3E-03	0.14
169	3.9E+03	1.1E+00	2.3E-08	1.3E-06	0.14
123	3.9E+00	1.1E-03	2.3E-05	1.3E-03	0.14
118	3.9E+00	1.1E-03	2.3E-05	1.3E-03	0.14
105	3.9E+00	1.1E-03	2.3E-05	1.3E-03	0.14
114	3.9E+00	1.1E-03	2.3E-05	1.3E-03	0.14
126	1.3E+04	3.8E+00	7.0E-09	4.0E-07	0.14
77	1.3E+01	3.8E-03	7.0E-06	4.0E-04	0.14
81	3.9E+01	1.1E-02	2.3E-06	1.3E-04	0.14
Non DL-PCBs	2.0E+00	5.7E-04			0.14

Abbreviation: Non DL-PCBs: Non-dioxin-like polychlorinated biphenyls

Note: Reference dose (RfD): estimate (with uncertainty spanning perhaps an order of magnitude) of daily oral exposure to the human population (including sensitive subgroups) that is likely to be without an appreciable risk of deleterious effects during a lifetime.^51^ Reference concentration (RfC): estimate (with uncertainty spanning perhaps an order of magnitude) of continuous inhalation exposure to the human population (including sensitive subgroups) that is likely to be without appreciable risk of deleterious effects during a lifetime.^51^

**Table 3:** Variables Used to Estimate Lifetime Cancer Risk^23^, ^51^–^52^, ^55^–^59^

**Exposure factor**	**Unit**	**Point estimate**
Ingestion rate (IR)^23^,^55^	(mg/day)	100
Exposed skin area (SA)^58^	(cm^2^)	3300
Skin adherence factor (AF_soii_)^57^	(mg/cm^2^)	0.2
Exposure frequency (EF)	(days/year)	313
Exposure duration (ED)^56^	(year)	25
Exposure time (ET)^51^	(h/day)	8
Body weight (BW)^52^	(kg)	60
Averaging time (AT) = (70 years × 365 days/year)^59^	(days)	25,550
Averaging time AT^*^ = (70 years × 365 days/year × 24 h/day)^51^	(h)	613,200

As shown in Table 3, the default value of ED of the USEPA is 25 years for outdoor workers.^56^ However, there is currently no data for Ethiopia. Exposure duration is estimated as the tenure of worker employment and exposure to non-residential soil.^56^ The present study used an estimated ED of 25 years and an EF of 313 days/year for workers based on respondent responses. In 2009, the USEPA published an updated handbook on exposure factors for human health risk assessments of soil ingestion rates (IngR) and 100 mg/day was recommended for adults based on an exposure duration ED of 70 years of lifetime exposure.^23^,^55^ Average working time has been estimated as 25,550 days [70 years × 365 days/year].^59^ The daily ET of workers was 8 h/day for continuous chronic exposure when assessing the risk posed by soil via inhalation.^54^ In addition, 613,200 h [70 years old × 365 days/year × 24 h/day] was the estimated average working time (AT*). The recommended BW of the local population for an adult worker was 60 kg,^52^ and contact SA of skin with soil was 3300 cm^2^, and the skin adherence factor of soil (AFsoil) was 0.2 mg/cm^2^ for workers.^57^, ^58^

## Results

The study questionnaire had a 99.6% respondent rate. The mean age of participants was 37.8 ± 2.5 years with a range from 19 to 63 years. Participants were fairly balanced between male (52.6%) and female (47.4%). With regard to education, 13.6% of respondents had a high school education, 66% had certificates, 26.1% had diplomas, and 35.2% had obtained at least a university degree.

### Workers' perception of occupational risk associated with use, maintenance and disposal of PCBs

The present study assessed the knowledge and level of awareness of workers handling PCBs and related materials. The results found that 48.8% of workers who have been engaged in the refurbishment and maintenance of transformers used personal protective equipment (PPE) such as gloves (35.5%), safety shoes and boots (21.5%) and masks (43%). Conversely, 51.2% indicated that they did not use safety equipment because of lack of availability (63.7%) or lack of awareness (36.3%). The storekeepers interviewed in this study had not received any form of training either on the management of PCBs or on the associated environmental health impacts of PCBs. The participants viewed oil and transformers containing PCBs as common harmless oil and equipment that did not pose any risk to their health. A male storekeeper interviewee from the transformer dumpsite related: *“I have not received any training since I started working here. I do not know if the oil and fluid we are working with is harmful or not to our health”.*

Workers were asked if they were trained on the management and environmental health effects of PCBs. Some of them (22.4%) stated that they had received training, whereas most (82.6%) had not. The results showed that 12.1% of the respondents stated that the dump site is in good condition, 44.8% stated that the dump site is illegal, and 43.1% had no knowledge of the conditions of the dump sites.

### Polychlorinated biphenyl-related accidents and public perception of PCB impacts

The present study investigated PCB accidents and participants' opinions on the possible impact of PCBs at selected sites. A total of 24.9% of participants reported that they had observed accidents that involved workers, while 75.1% reported that had not observed any accidents. Participants noted that groundwater, air, and soil were the most affected media, at 13.2%, 11.8%, and 10%, respectively. About 65% of participants responded that if not properly managed, PCBs in oil and transformers could pollute environmental media. In addition, 33.7% of respondents claimed that they were affected by bad odor, and 31.2% reported general illness such as headache, coughing, and nausea as a result of improper storage of PCB-containing materials. In addition to the pungent smell and health complications noted by some respondents, 35.1% of participants reported that dump site was unaesthetic.

### Chi-square test and cross tabulation

Around one-third of both males (31%) and females (31.5%) perceived the impact of PCBs on human health and the environment *(Table 4).* Respondents who had obtained at least a bachelor's degree and above (30.6%) perceived that PCBs had a negative impact on the environment as well as humans.

**Table 4:** Distribution of Perception of the Impact of PCBs on Humans and the Environment with Sociodemographic Characteristics

		**Perception of impact of PCBs on human health and environment**	

		**Yes**	**No**
Sex	Male	82 (31%)	58 (22%)
	Female	83 (31.5%)	41 (15.5%)

Education level	Secondary school	21 (8%)	12 (4.5%)
	Certificate (10+1)	44 (16.7%)	22 (8.4%)
	Diploma (10+3)	53 (20.1%)	19 (7.1%)
	First degree and above	81 (30.6%)	12 (4.5%)

Abbreviation: PCBs-Polychlorinated biphenyls

Table 5 presents the perception of respondents of the impact of PCBs with respect to related occupational exposure variables. Out of the nearly 83% of study participants who stated they had witnessed oil spillage in the study area, 73.3% perceived this impact as negative. Pearson's chi-squared test (p < 0.05) was used to assess workers' perception of occupational safety issues associated with work at the study sites. Workers who used PPE, were trained on the impact of hazardous chemicals, and observed the leakage of PCB-containing oil perceived that the site is not well protected. These workers were less likely to experience or consciously be aware of the effects of PCBs on humans and the environment.

**Table 5:** Perception of the Association of the Impact of PCBs on Humans and the Environment Across Occupational Exposure Variables

**Variables**		**Perception of impact of PCBs on human health and the environment**			

		**Yes**	**No**	**χ^2^ value**	**P value**
Use of personal protective equipment	Yes	78 (29.5%)	51 (19.3%)	49.15	0.000**
	No	36 (13.6%)	99 (37.6%)		
Training on hazardous chemicals	Yes	51 (19.3%)	5 (1.8%)	32.58	0.031**
	No	105 (39.8%)	103 (39.1%)		
Leakage of oil from pipeline	Yes	129 (48.9%)	94 (35.6%)	7.85	0.026**
	No	11 (4.1%)	30 (11.4%)		
Spillage of oil in workshop areas	Yes	195 (73.9%)	22 (8.4)	3.17	0.087
	No	8 (3%)	39 (14.7%)		
Presence of other electric industries in study area	Yes	116 (43.9%)	65 (24.6%)	2.11	0.152
	No	51 (19.3%)	32 (12.1%)		
Emissions from workshop	Yes	148 (46%)	43 (16.3%)	1.98	0.243
	No	46 (17.4%)	27 (10.2%)		
Dump site protection	Yes	59 (22.3%)	32 (12.1)	27.43	0.048**
	No	139 (52.7%)	34 (12.9%)		

Abbreviation: PCBs-Polychlorinated biphenyls

** Significant difference at *p* < 0.05

### Health risk assessment

The human health risk assessment is a useful tool to measure the probability of health impacts of environmental chemicals on humans due to chemical exposure.^57^ The current study estimated the potential health risks of an adult worker due to exposure to 12 DL and six (6) non DL-PCBs via accidental ingestion of soil particles, dermal contact pollutant absorption, and inhalation of fugitive soil particles *(Tables 6*–*8).* The carcinogenic risks of different land uses for PCBs combining the above exposure pathways are shown in Table 9. The average values of the health risks of dermal contact, accidental ingestion, and inhalation were 1.17 × 10^−3^ (range from 1.75 × 10^−06^ to 4.822 × 10^−3^), 1.6 × 10^−5^ (ranging from 3.45 ×10^−07^ to 4.19 ×10^−05^), and 2.33 ×10^−11^ (ranging from 7.16 ×10^−12^ to 1.54 × 10^−10^), respectively.

**Table 6:** Lifetime Cancer Risk via Dermal Contact/Absorption Pathway Across Study Sites

**PCB**	**S1**	**S2**	**S3**	**S4**	**S5**	**S6**	**S7**	**S8**	**S9**
28	**1.0E-05**	9.9E-07	**1.1E-06**	**1.1E-06**	9.1E-07	**2.2E-06**	**2.1E-06**	2.1E-07	**2.3E-06**
51	**7.9E-06**	**1.1E-06**	7.1E-07	7.1E-07	7.0E-07	7.9E-07	**2.1E-06**	5.4E-07	8.2E-07
77	**4.0E-06**	**5.5E-06**	**4.5E-06**	**3.1E-06**	**2.4E-06**	**3.4E-06**	**2.1E-06**	**2.2E-06**	**5.5E-06**
81	**1.6E-05**	**1.9E-05**	**1.3E-05**	**1.7E-05**	**1.1E-05**	**1.9E-05**	**8.9E-06**	**7.4E-06**	**1.6E-05**
101	3.6E-07	9.1E-08	6.5E-08	6.5E-08	1.4E-08	9.4E-08	3.6E-08	2.8E-08	1.9E-08
105	0.0E+00	1.5E-07	2.7E-07	**1.7E-06**	1.1E-07	**2.4E-06**	6.0E-07	2.3E-08	2.1E-07
114	3.2E-07	2.1E-07	1.5E-07	**1.8E-06**	9.9E-08	5.7E-07	1.5E-07	1.1E-07	2.2E-07
118	2.4E-07	4.0E-08	1.1E-07	**1.7E-06**	6.8E-08	0.0E+00	2.3E-07	2.3E-07	5.7E-08
123	1.4E-07	1.6E-07	3.1E-07	**2.7E-06**	2.3E-07	7.6E-07	4.6E-07	2.2E-07	2.0E-07
126	0.0E+00	**7.8E-04**	**2.3E-03**	**3.7E-03**	**1.3E-03**	**1.2E-03**	**6.7E-04**	8.9E-04	**2.5E-03**
138	1.2E-07	2.7E-07	2.3E-08	7.1E-07	1.5E-07	4.4E-07	2.1E-07	1.4E-07	7.3E-08
153	3.1E-07	2.3E-07	5.2E-08	3.5E-07	2.1E-07	2.0E-07	1.2E-07	1.6E-07	1.8E-07
156	3.7E-07	1.8E-07	2.5E-07	3.4E-07	7.9E-08	5.0E-07	7.0E-07	7.0E-07	**1.3E-06**
157	4.2E-07	1.1E-07	1.7E-08	3.0E-07	4.8E-07	7.1E-07	2.2E-07	**1.3E-06**	**1.7E-06**
167	3.4E-07	2.5E-07	1.1E-07	**1.2E-06**	5.9E-08	6.5E-07	5.7E-07	**1.2E-06**	5.9E-07
169	**8.5E-04**	**4.7E-04**	**6.9E-04**	**8.0E-04**	**1.7E-04**	6.7E-04	**8.8E-04**	**8.1E-04**	2.3E-03
180	0.0E+00	0.0E+00	2.0E-08	1.1E-07	1.9E-07	2.0E-07	1.1E-07	6.8E-07	8.7E-07
189	**1.5E-06**	**1.5E-06**	**1.2E-06**	1.9E-07	**1.5E-06**	1.7E-08	3.8E-08	5.4E-08	1.6E-07

Total risk	9.0E-04	1.3E-03	3.0E-03	4.5E-03	1.4E-03	1.9E-03	1.6E-03	1.7E-03	4.8E-03

Abbreviation: PCB-Polychlorinated biphenyl

Note: **Bold** indicates potential cancer risk

**Table 7:** Lifetime Cancer Risk via Accidental Ingestion Pathway Across Study Sites

**PCB**	**S1**	**S2**	**S3**	**S4**	**S5**	**S6**	**S7**	**S8**	**S9**
28	**2.0E-06**	2.0E-07	2.1E-07	2.1E-07	1.8E-07	4.3E-07	4.2E-07	4.1E-08	4.6E-07
51	**1.6E-06**	2.1E-07	1.4E-07	1.4E-07	1.4E-07	1.6E-07	4.0E-07	1.1E-07	1.6E-07
77	**3.1E-06**	**3.7E-06**	**2.6E-06**	**3.4E-06**	**2.3E-06**	**3.8E-06**	**1.8E-06**	**1.4E-06**	**3.1E-06**
81	**3.1E-06**	**3.7E-06**	**2.6E-06**	**3.4E-06**	**2.3E-06**	**3.8E-06**	**1.8E-06**	**1.4E-06**	**3.1E-06**
101	7.2E-08	1.8E-08	1.3E-08	1.3E-08	2.8E-09	1.8E-08	7.0E-09	5.5E-09	3.8E-09
105	**3.1E-06**	**3.7E-06**	**2.6E-06**	**3.4E-06**	**2.3E-06**	**3.8E-06**	**1.8E-06**	**1.4E-06**	**3.1E-06**
114	**3.1E-06**	**3.7E-06**	**2.6E-06**	**3.4E-06**	**2.3E-06**	**3.8E-06**	**1.8E-06**	**1.4E-06**	**3.1E-06**
118	**3.1E-06**	**3.7E-06**	**2.6E-06**	**3.4E-06**	**2.3E-06**	**3.8E-06**	**1.8E-06**	**1.4E-06**	**3.1E-06**
123	**3.1E-06**	**3.7E-06**	**2.6E-06**	**3.4E-06**	**2.3E-06**	**3.8E-06**	**1.8E-06**	**1.4E-06**	**3.1E-06**
126	**3.1E-06**	**3.7E-06**	**2.6E-06**	**3.4E-06**	**2.3E-06**	**3.8E-06**	**1.8E-06**	**1.4E-06**	**3.1E-06**
138	2.4E-08	5.3E-08	4.5E-09	1.4E-07	2.9E-08	8.7E-08	4.1E-08	2.7E-08	1.4E-08
153	6.0E-08	4.5E-08	1.0E-08	6.9E-08	4.1E-08	4.0E-08	2.4E-08	3.2E-08	3.6E-08
156	**3.1E-06**	**3.7E-06**	**2.6E-06**	**3.4E-06**	**2.3E-06**	**3.8E-06**	**1.8E-06**	**1.4E-06**	**3.1E-06**
157	**3.1E-06**	**3.7E-06**	**2.6E-06**	**3.4E-06**	**2.3E-06**	**3.8E-06**	**1.8E-06**	**1.4E-06**	**3.1E-06**
167	**3.1E-06**	**3.7E-06**	**2.6E-06**	**3.4E-06**	**2.3E-06**	**3.8E-06**	**1.8E-06**	**1.4E-06**	**3.1E-06**
169	**3.1E-06**	**3.7E-06**	**2.6E-06**	**3.4E-06**	**2.3E-06**	**3.8E-06**	**1.8E-06**	**1.4E-06**	**3.1E-06**
180	0.0E+00	0.0E+00	4.0E-09	2.2E-08	3.7E-08	4.0E-08	2.1E-08	1.3E-07	1.7E-07
189	3.0E-07	2.9E-07	2.4E-07	3.7E-08	3.0E-07	3.4E-09	7.5E-09	1.1E-08	3.2E-08

Total risk	3.8E-05	4.2E-05	2.9E-05	3.8E-05	2.6E-05	4.3E-05	2.0E-05	1.6E-05	3.5E-05

Abbreviation: PCB-Polychlorinated biphenyl

Note: **Bold** indicates potential cancer risk

**Table 8:** Lifetime Cancer Risk Via Inhalation Exposure Pathway Across Study Sites

**PCB**	**S1**	**S2**	**S3**	**S4**	**S5**	**S6**	**S7**	**S8**	**S9**
28	8.2E-11	8.2E-12	8.8E-12	8.8E-12	7.5E-12	1.8E-11	1.8E-11	1.7E-12	1.9E-11
51	6.5E-11	8.8E-12	5.9E-12	5.9E-12	5.8E-12	6.5E-12	1.7E-11	4.4E-12	6.8E-12
77	3.4E-14	3.4E-14	3.4E-14	3.4E-14	3.4E-14	3.4E-14	3.4E-14	3.4E-14	3.4E-14
81	1.3E-13	1.3E-13	1.3E-13	1.3E-13	1.3E-13	1.3E-13	1.3E-13	1.3E-13	1.3E-13
101	3.0E-12	7.5E-13	5.3E-13	5.3E-13	1.2E-13	7.7E-13	2.9E-13	2.3E-13	1.6E-13
105	0.0E+00	1.2E-15	2.2E-15	1.4E-14	9.2E-16	2.0E-14	4.9E-15	1.9E-16	1.7E-15
114	2.6E-15	2.6E-15	2.6E-15	2.6E-15	2.6E-15	2.6E-15	2.6E-15	2.6E-15	2.6E-15
118	1.9E-15	1.9E-15	1.9E-15	1.9E-15	1.9E-15	1.9E-15	1.9E-15	1.9E-15	1.9E-15
123	1.1E-15	1.1E-15	1.1E-15	1.1E-15	1.1E-15	1.1E-15	1.1E-15	1.1E-15	1.1E-15
138	1.0E-12	2.2E-12	1.9E-13	5.8E-12	1.2E-12	3.6E-12	1.7E-12	1.1E-12	6.0E-13
153	2.5E-12	1.9E-12	4.3E-13	2.9E-12	1.7E-12	1.7E-12	1.0E-12	1.3E-12	1.5E-12
126	0.0E+00	6.6E-12	1.9E-11	3.1E-11	1.1E-11	1.0E-11	5.6E-12	7.5E-12	2.1E-11
156	3.0E-15	3.0E-15	3.0E-15	3.0E-15	3.0E-15	3.0E-15	3.0E-15	3.0E-15	3.0E-15
157	3.4E-15	3.4E-15	3.4E-15	3.4E-15	3.4E-15	3.4E-15	3.4E-15	3.4E-15	3.4E-15
167	2.8E-15	2.8E-15	2.8E-15	2.8E-15	2.8E-15	2.8E-15	2.8E-15	2.8E-15	2.8E-15
180	0.0E+00	0.0E+00	1.7E-13	9.1E-13	1.5E-12	1.7E-12	9.0E-13	5.6E-12	7.2E-12
169	7.0E-12	7.0E-12	7.0E-12	7.0E-12	7.0E-12	7.0E-12	7.0E-12	7.0E-12	7.0E-12
189	1.3E-14	1.3E-14	1.3E-14	1.3E-14	1.3E-14	1.3E-14	1.3E-14	1.3E-14	1.3E-14

Total risk	1.6E-10	3.6E-11	4.3E-11	6.3E-11	3.6E-11	5.0E-11	5.1E-11	2.9E-11	6.4E-11

Abbreviation: PCB-Polychlorinated biphenyl

**Table 9:** Human Cancer Risk in Humans via Ingestion, Dermal Contact and Inhalation of Soil Particles Across Sampling Sites

**Sampling sites**	**Cancer risk via ingestion**			**Cancer risk via dermal contact**			**Cancer risk via inhalation**		

	**10^th^ Centile**	**Median**	**95^th^ Centile**	**10^th^ Centile**	**Median**	**95^th^ Centile**	**10^th^ Centile**	**Median**	**95^th^ Centile**
1	2.392E-05	**3.116E-03**	**3.116E-03**	0	3.54E-04	**0.854**	0	8.021E-12	8.229E-08
2	1.780E-05	**3.739E-03**	**3.739E-03**	3.96E-05	2.4E-04	**0.468**	6.609E-09	2.315E-11	8.191E-09
3	4.547E-06	**2.617E-03**	**2.617E-03**	2.03E-05	2.57E-04	**0.685**	1.937E-08	8.147E-11	8.803E-09
4	2.173E-05	**3.383E-03**	**3.383E-03**	1.10E-04	1.14E-04	**0.803**	3.139E-08	8.147E-11	8.803E-09
5	2.949E-05	**2.261E-03**	**2.261E-03**	5.93E-05	2.17E-04	**0.171**	1.062E-08	7.463E-11	7.463E-09
6	1.844E-05	**3.810E-03**	**3.810E-03**	1.73E-05	6.78E-04	**0.667**	1.031E-08	8.147E-11	1.810E-08
7	7.515E-06	**1.759E-03**	**1.759E-03**	3.82E-05	5.17E-04	**0.879**	5.639E-09	8.147E-11	1.756E-08
8	1.056E-05	**1.447E-03**	**1.447E-03**	2.79E-05	3.86E-04	**0.081**	7.502E-09	8.147E-11	1.703E-09
9	1.442E-05	**3.080E-03**	**3.080E-03**	5.74E-05	7.06E-04	2.316	2.098E-08	8.147E-11	1.932E-09

Note: **Bold** indicates potential cancer risk [all cancer risks are presented in units of 10^−3^ except 0 values at the 10^th^ centile]

In Table 6, the possibility of lifetime cancer risk was estimated and ranged from high to moderate. However, the total lifetime cancer risk per site was high. The probability of developing cancer via dermal contact at the swamp sample site (S9) was the highest at 4.8E-3, followed by the transformer area (S4) at 4.5E-3, whereas the oil tanker and oil barrel sites had a moderate level of risk [9.0E-04].

In Table 7, the estimated lifetime cancer risk for all sample sites was low (ranging from 10^−6^ < to <10^−4^) for the accidental ingestion route. However, at the workshop area (S6), the estimated lifetime cancer risk was higher. The estimated lifetime cancer risk was lower at the Civil Service University student dormitory site (S8) compared to the other sampling sites.

As seen in Table 8, the lifetime cancer risk across all samples sites was very low (10^−6^) via the inhalation route.

As seen in Table 9, the cancer risk values from the PCB-contaminated soils across all nine study sites for dermal contact were below 1 in 1000 at the 5th and 95th percentiles, indicating that the cancer risks imposed by these 18 PCBs are high. However, when considering inhalation as an exposure pathway, the cancer risk due to inhalation of soil particles for all sites was below 1 in 1 000 000 000 at the 5^th^, 50^th^ and 95^th^ percentiles, indicating that it poses a very low cancer risk (less than 1 in a million or <10^−6^). At the 10^th^ percentile, in the case of the other three land use types, the cancer risk trend was as follows: S4 > S9 > S3 for dermal contact pathways. At the 50th and 95th percentile, all types of land use registered very low cancer risk via the ingestion pathway.

## Discussion

The Stockholm convention on POPs, of which Ethiopia is a party and signatory country, emphasized the need for capacity building, awareness raising, and training materials for managing chemicals such as PCBs.^62^ Implementation of these recommended guidelines is a challenge in Ethiopia. The questionnaire analysis showed that workers who were aware of health risks from exposure to PCBs perceived that exposure to PCBs could result in detrimental human and environmental consequences. This indicates that those who had been trained on PCB risk are more cautious and able to protect themselves than those without risk information.^63^ Nevertheless, it is not always true that those with a better understanding of health impacts understand the health effects of PCBs. Sometimes people's perception and level of understanding depend on daily experience and personal reading habits and mass media influences.

The unavailability of PPE for workers is a cause for concern as oils from transformers normally contains high concentrations of PCBs. For example, a study in Delta State, Nigeria found that the Σ14 PCB concentrations in transformer, turbine and compressor oils ranged from 484 to 48,506 mg kg−1, which was several thousand-fold higher than the concentrations recorded for the other environmental media around the power plant. Another study in China found 63% of tri-PCBs, 24% of tetra-PCBs and 9% of di-PCBs in transformer oil.^56^

We found that poor handling and storage of equipment containing PCBs was largely due to low level of awareness of workers of occupational safety procedures. A report from Tanzania indicated that due to low awareness, workers were exposed to PCB-contaminated oil without use of PPE.^64^ In Ethiopia, training and capacity building programs for workers on occupational health and safety is an issue often overlooked by policy and programs on POPs, including PCBs. The open dumping of transformers and other materials containing PCBs have potential aesthetic, health, and environmental impacts. Illegal dumping of electronic wastes containing PCBs is common in many African countries.^64^ This practice of open dumping of PCBs has both short- and long-term environmental and health implications.

### Polychlorinated biphenyl-related accidents and workers' perception of PCB impacts

The majority of respondents (74%) had not witnessed any accidents in the study areas. Length of employment may have affected these results. Accidents in this type of sector are likely to occur during the process of refueling of oil to refurbished transformers. About 65% of participants responded that if not properly managed, PCBs in oil and transformers could pollute environmental media. This is an agreement with the findings of other studies which noted that PCBs can contaminate all environmental media.^65^ Some (33.7%) of the respondents stated that the study area was not properly managed.^44^ If chemicals are not properly stored, they could produce malodors and other hazards, as seen in a study in China.^66^ Improper management of scrap transformers and capacitors has impacted the quality of the environment at the study sites due to the possibility of contamination of surrounding environmental media.^44^ The overflow of oil from tankers and oil leakage from transformers has the potential to pollute the environment as well. The participants' level of education could influence their level of perception of the environmental and health implications of PCBs.

### Health risk assessment

Cancer risk calculations were used for screening purposes only and are interpreted as preliminary indications of potential cancer risks. Polychlorinated biphenyls are carcinogenic to living organisms and humans could be exposed to PCB-contaminated soils via different exposure pathways.^67^ The present study indicated that workers who were most at risk of PCB exposure were those working at the maintenance workshop site.

The cancer risk of PCBs for the above-mentioned exposure routes increased in the order of inhalation < ingestion < dermal contact. Dermal contact is a significant exposure pathway for PCBs. This may be due to the infrequent usage of PPE. This indicates that the health risk due to the inhalation pathway may be negligible in the present study. The order of the exposure pathways is due to the low volatility of PCBs.^67^ The estimated cancer risk for PCBs via dermal contact was relatively higher than the accidental ingestion and inhalation pathways. This result is contrary to a recent study that reported the risk of PCB exposure in soil from the ingestion pathway surpassed that from dermal contact and inhalation.^35^ The health risk associated with dermal contact in our study was 73.79 times higher than the inhalation exposure routes. This could indicate that the health risk due to inhalation may be negligible in this study. The estimated inhalation cancer risk for PCBs was very low compared to other exposure routes (dermal contact and ingestion). However, this may be a consequence of the lack of full evaluation of air samples in the present study.^52^ The estimated cancer risks via inhalation should be based on pollutants adsorbed onto respirable particles of soils (less than PM_10_).^68^ Only inhaled soil particles smaller than PM_10_ can be deposited in the upper part of the respiratory tract and/or penetrate deeply into the lungs.^52^,^69^ Fine soil particles with organic pollutants such as PCBs may be able to cause stress and inflammation after penetrating the lung.^70^ Concentrations of pollutants in soil particles with a diameter of less than 2 mm should be lower than for particles smaller than PM_10_.^52^,^71^

Dermal absorption is a significant exposure pathway for PCBs at the transformer maintenance workshop site (S6). As previously mentioned, the cancer risk for PCBs via dermal contact was relatively high compared to accidental ingestion and inhalation. Our results are in agreement with a study conducted in Hong Kong that reported that dermal contact was the main exposure route of PCBs for electronic waste workers.^52^,^72^ The average values of the health risk of dermal contact, accidental ingestion, and inhalation were 1.17 × 10^−3^, 1.6 × 10^−5^, and 2.33 × 10^−11^, respectively. This shows that the risk is primarily from dermal contact and accidental ingestion and a similar assertion had been reported in a study in China.^73^

#### Cancer risk of dioxin-like PCBs and non-dioxin-like PCBs via different exposure pathways

The cancer risks of DL-PCBs were estimated on the basis of three exposure routes *(Supplemental Material 2, Tables 1, 2 and 3).* Table 6 indicates that the cancer risk via dermal contact across all study sites was high with the highest risk value of 4.82 × 10^−03^ at the site situated in a swamp area. The cancer risk showed a moderate level (8.78 × 10^−4^) at the oil tanker and barrel site with an average of 2.34 × 10^−3^. The estimated lifetime cancer for ingestion routes of each site was low and ranged from 1.59 × 10^−5^ to 4.19 × 10^−5^ with an average of 3.1 × 10^−5^. Dermal contact and accidental ingestion are the routes with the highest chance of workers developing cancer. The tested samples taken from all sites had a very low carcinogenic risk for the inhalation exposure route with an average of 7.16 × 10^−12^. The estimated cancer risk via inhalation was based on pollutant adsorption of a respirable particle of soils less than PM_10_.^58^ According to previous studies, inhaled soil particles less than PM_10_ can accumulate in the upper part of the respiratory tract and penetrate deeply into the lung.^72^–^74^ For non-DL PCBs, the cancer risk via dermal contact ranged from 1.75 × 10^−6^ to 1.87 × 10^−5^ with an average of 4.79 × 10^−6^; the cancer risk for accidental ingestion ranged from 3.45 × 10^−7^ to 3.68 × 10^−6^ with an average of 9.43 × 10^−7^; and the cancer risk for inhalation ranged from 1.45 × 10^−11^ to 1.54 × 10^−10^ with an average of 3.95 × 10^−11^ *(Supplemental Material 2Tables 4, 5 and 6 ).*

Most of the reviewed studies indicated that the excess lifetime cancer risk (≥10^−6^) was considered to be insignificant, with excess lifetime cancer risks of ≥10^−4^ considered to be significant.^53^,^75^ There is no consistency in interpreting cancer risks between 10^−6^ and 10^−4^ and the actions taken to reduce these risks varies from one country to another. For instance, the Canadian soil quality guidelines state that a cancer risk of ≤10^−5^ is considered to be insignificant. One of the best approaches to lowering the cancer risk stemming from contaminated land is to remediate the soil, an approach that is recommended in the present study. Recent advances in bioremediation of PCBs in soil have been well documented.^76^

One limitation of the present study was that exposure routes were estimated by the default values across exposures.^52^,^72^,^77^ Estimation of cancer risk using the default value may lead to overestimation of results. In addition, the estimated cancer risk reported in the current study was calculated based on exposure to only one chemical with different congeners, whereas humans are exposed to mixtures of many carcinogenic chemicals. Another limitation of the current study was the use of soil particles of less than 2 mm for the risk assessment evaluation via the inhalation pathway instead of air samples of respirable particles.

## Conclusions

The present study provides preliminary data on occupational health and safety issues related to the management of PCBs in Ethiopia. The present study explored important factors associated with the likelihood of cancer risk for workers who have been occupationally exposed to PCBs. The results obtained from this study revealed 51.2% of the study participants did not use PPE and 82% were not trained in the handling and safe disposal of PCBs, and as such were unaware of the potential effects of these hazardous chemicals. The potential cancer risk of PCBs was calculated based on the concentration data obtained in previous study. The results revealed that the cancer risk ranged from very low to high across all exposure pathways. Dermal absorption of PCBs is the major route of exposure followed by ingestion, with an average estimated lifetime cancer risk of 2.34 × 10^−3^ and 3.1 × 10^−^5, respectively. The calculated inhalation potential cancer risks are very low compared to other exposure routes. This may be due to a lack of air samples of respirable particles (PM_10_), and future studies should include air sampling to estimate potential cancer risk via the inhalation route.

The findings of the present study indicate the need to improve the safety of workers handling transformers, capacitors and other e-waste-containing PCBs to minimize the potential cancer risk for workers. Furthermore, training of workers on safety awareness and safety behavior is key to reducing the rate of work-related accidents and unsafe practices. Therefore, it is important to initiate focused and targeted programs geared towards strengthening of human resources and the capacity of worker in order to reduce occupational PCBs exposure as well as promoting awareness.

There are currently no regulations on the safe management of PCB-contaminated soils in Ethiopia. As the present study indicated potential cancer risks due to exposure to PCB-contaminated soil, guidelines or standards for PCBs in soil are needed in order to protect human health. In addition, the Stockholm Convention and the United Nations Industrial Development Organization (UNIDO) should closely follow the status of the national PCB contamination hotpots, and offer measures that could assist governments and other authorities to better manage PCB-containing materials and equipment in Ethiopia. For example, with support from the United Nations Industrial Development Organization (UNIDO) and the Global Environment Facility (GEF), a PCB destruction facility was set up in the Philippines with the capacity to minify PCB oils (< 10,000 mg/kg) to the Philippine guideline standards of less than 2.0 mg/kg.^78^ The replication of such initiatives is urgently needed in Ethiopia.

While this study has shed light on the cancer risk of workers exposed to PCBs, more data are needed to understand other potential health implications, including the types of cancer associated with exposure to these chemicals. Thus, an interdisciplinary approach employing epidemiology study designs will provide a better fundamental understanding of PCB levels in serum and the full health impacts of PCBs in humans.
