# Supplementary material for: Assessment of Perceptions and Cancer Risks of Workers at a Polychlorinated Biphenyl-Contaminated Hotspot in Ethiopia
Source: J Health Pollut. 2021 May 28;11(30):210609. doi: 10.5696/2156-9614-11.30.210609 (PMC8276727; doi:10.5696/2156-9614-11.30.210609)
Supplement: Supplementary file 1 [file Debela_Supplemental_Material_1.docx]

**Supplemental Material**

**Questionnaire**

**Perception and Cancer Risk Assessment of Workers at a National Polychlorinated Biphenyl-Contaminated Hotspot in Ethiopia**

Code.______

Questionnaire for **Perception and Cancer Risk Assessment of Workers at a National Polychlorinated Biphenyl-Contaminated hotspot in Ethiopia.**

.
Hello, I am__________________.

I would like to ask you a few questions about the Kotobe transformer maintenance and workshop and dump site. This will help us to improve occupational safety, health and working environment services provided to you based on your answer to our questions. Your name will not be written in this form and will never be used in connection with any information you tell us. All information given by you will be kept strictly confidential. Your participation is voluntary and you are not obliged to answer any question you do not wish to answer. If you feel uncomfortable with the interview please feel free to drop out of the study any time you want. This interview will take about 30 minutes. Do I have your permission to continue?

1. If yes, continue to the next page
2. If no, skip to the next participant after writing reasons for his/her refusal

Informed consent certified by
Interviewer: Code_____________ Name__________________________ Signature________
Date of interview ____________Time started ___________ Time completed________
Result of interview: 1.Completed 2. Respondent not available 3. Refused 4. Partially completed

Checked by Supervisor: Name_____________ Signature ___________ Date_______

Educational status: _____________________

Gender: ____________________________

Age: ____________________________

Contact number: ___________________

1. Is there any chemical spillage in the local workshop?

□Yes □No

2. Is any transformer effluent generated from the workshop?

□Yes □No

3. Are there emissions during maintenance of the workshop?

□Yes □No

3. Has anyone in the local area ever smelled unusual odors from the soil?

□Yes □No

4. Do you use safety equipment for your workshop activities?

□Yes □No

5. If the answer for Q4 is “Yes”, what safety materials do you use?

A. Gloves B. Masks C. Safety shoes D. All E------

6. If the answer for Q4 is “No”, what is/are your reason(s)?

A. Lack of safety equipment B. Lack of awareness

7. Did you train on the impact of hazardous chemicals such as PCBs?

□Yes □No

8. Did you train on how to manage e-waste such as transformers?

□Yes □No

9. Are you aware of any risks posed by PCB-containing transformers?

□Yes □No

10. If the answer for Q9 is “Yes”, What are the possible risks/impacts to people?

A. Illness B. Pungent odor C. Environmental pollution

D. If others-----------

11. Do you know if the site is protected?

□Yes □No

12. Did you observed any incidents in the last year?

□ Yes □No

13. Have you ever observed overflow of PCB-containing oil in the workshop?

□Yes □No
