# Supplementary material for: Assessment of Perceptions and Cancer Risks of Workers at a Polychlorinated Biphenyl-Contaminated Hotspot in Ethiopia
Source: J Health Pollut. 2021 May 28;11(30):210609. doi: 10.5696/2156-9614-11.30.210609 (PMC8276727; doi:10.5696/2156-9614-11.30.210609)
Supplement: Supplementary file 2 [file Debela_Supplemental_Material_2.docx]

**Supplemental Material 2**

Table 1: Calculated Lifetime Cancer Risk via Ingestion for Dioxin-like Polychlorinated Biphenyls Across Study Sites

| **PCB** | **S1** | **S2** | **S3** | **S4** | **S5** | **S6** | **S7** | **S8** | **S9** |
| --- | --- | --- | --- | --- | --- | --- | --- | --- | --- |
| 81 | 3.10E-06 | 3.70E-06 | 2.60E-06 | 3.40E-06 | 2.30E-06 | 3.80E-06 | 1.80E-06 | 1.40E-06 | 3.10E-06 |
| 77 | 3.10E-06 | 3.70E-06 | 2.60E-06 | 3.40E-06 | 2.30E-06 | 3.80E-06 | 1.80E-06 | 1.40E-06 | 3.10E-06 |
| 105 | 3.10E-06 | 3.70E-06 | 2.60E-06 | 3.40E-06 | 2.30E-06 | 3.80E-06 | 1.80E-06 | 1.40E-06 | 3.10E-06 |
| 114 | 3.10E-06 | 3.70E-06 | 2.60E-06 | 3.40E-06 | 2.30E-06 | 3.80E-06 | 1.80E-06 | 1.40E-06 | 3.10E-06 |
| 118 | 3.10E-06 | 3.70E-06 | 2.60E-06 | 3.40E-06 | 2.30E-06 | 3.80E-06 | 1.80E-06 | 1.40E-06 | 3.10E-06 |
| 123 | 3.10E-06 | 3.70E-06 | 2.60E-06 | 3.40E-06 | 2.30E-06 | 3.80E-06 | 1.80E-06 | 1.40E-06 | 3.10E-06 |
| 126 | 3.10E-06 | 3.70E-06 | 2.60E-06 | 3.40E-06 | 2.30E-06 | 3.80E-06 | 1.80E-06 | 1.40E-06 | 3.10E-06 |
| 156 | 3.10E-06 | 3.70E-06 | 2.60E-06 | 3.40E-06 | 2.30E-06 | 3.80E-06 | 1.80E-06 | 1.40E-06 | 3.10E-06 |
| 157 | 3.10E-06 | 3.70E-06 | 2.60E-06 | 3.40E-06 | 2.30E-06 | 3.80E-06 | 1.80E-06 | 1.40E-06 | 3.10E-06 |
| 167 | 3.10E-06 | 3.70E-06 | 2.60E-06 | 3.40E-06 | 2.30E-06 | 3.80E-06 | 1.80E-06 | 1.40E-06 | 3.10E-06 |
| 169 | 3.10E-06 | 3.70E-06 | 2.60E-06 | 3.40E-06 | 2.30E-06 | 3.80E-06 | 1.80E-06 | 1.40E-06 | 3.10E-06 |
| 189 | 3.00E-07 | 2.90E-07 | 2.40E-07 | 3.70E-08 | 3.00E-07 | 3.40E-09 | 7.50E-09 | 1.10E-08 | 3.20E-08 |
| Total | *3.50E-05* | *4.10E-05* | *2.90E-05* | *3.70E-05* | *2.50E-05* | *4.20E-05* | *1.90E-05* | *1.60E-05* | *3.40E-05* |

Table 2: Calculated Lifetime Cancer Risk via Dermal Contact for Dioxin-like Polychlorinated Biphenyls Across Study Sites

| **PCB** | **S1** | **S2** | **S3** | **S4** | **S5** | **S6** | **S7** | **S8** | **S9** |
| --- | --- | --- | --- | --- | --- | --- | --- | --- | --- |
| 81 | 1.60E-05 | 1.90E-05 | 1.30E-05 | 1.70E-05 | 1.10E-05 | 1.90E-05 | 8.90E-06 | 7.40E-06 | 1.60E-05 |
| 77 | 4.00E-06 | 5.50E-06 | 4.50E-06 | 3.10E-06 | 2.40E-06 | 3.40E-06 | 2.10E-06 | 2.20E-06 | 5.50E-06 |
| 105 | 0.00E+00 | 1.50E-07 | 2.70E-07 | 1.70E-06 | 1.10E-07 | 2.40E-06 | 6.00E-07 | 2.30E-08 | 2.10E-07 |
| 114 | 3.20E-07 | 2.10E-07 | 1.50E-07 | 1.80E-06 | 9.90E-08 | 5.70E-07 | 1.50E-07 | 1.10E-07 | 2.20E-07 |
| 118 | 2.40E-07 | 4.00E-08 | 1.10E-07 | 1.70E-06 | 6.80E-08 | 0.00E+00 | 2.30E-07 | 2.30E-07 | 5.70E-08 |
| 123 | 1.40E-07 | 1.60E-07 | 3.10E-07 | 2.70E-06 | 2.30E-07 | 7.60E-07 | 4.60E-07 | 2.20E-07 | 2.00E-07 |
| 126 | 0.00E+00 | 7.80E-04 | 2.30E-03 | 3.70E-03 | 1.30E-03 | 1.20E-03 | 6.70E-04 | 8.90E-04 | 2.50E-03 |
| 156 | 3.70E-07 | 1.80E-07 | 2.50E-07 | 3.40E-07 | 7.90E-08 | 5.00E-07 | 7.00E-07 | 7.00E-07 | 1.30E-06 |
| 157 | 4.20E-07 | 1.10E-07 | 1.70E-08 | 3.00E-07 | 4.80E-07 | 7.10E-07 | 2.20E-07 | 1.30E-06 | 1.70E-06 |
| 167 | 3.40E-07 | 2.50E-07 | 1.10E-07 | 1.20E-06 | 5.90E-08 | 6.50E-07 | 5.70E-07 | 1.20E-06 | 5.90E-07 |
| 169 | 8.50E-04 | 4.70E-04 | 6.90E-04 | 8.00E-04 | 1.70E-04 | 6.70E-04 | 8.80E-04 | 8.10E-04 | 2.30E-03 |
| 189 | 1.50E-06 | 1.50E-06 | 1.20E-06 | 1.90E-07 | 1.50E-06 | 1.70E-08 | 3.80E-08 | 5.40E-08 | 1.60E-07 |
| Total | *8.80E-04* | *1.30E-03* | *3.00E-03* | *4.50E-03* | *1.40E-03* | *1.90E-03* | *1.60E-03* | *1.70E-03* | *4.80E-03* |

Table 3: Calculated Lifetime Cancer Risk via Inhalation for Dioxin-like Polychlorinated Biphenyls Across Study Sites

|  |  |  |  |  |  |  |  |  |  |
| --- | --- | --- | --- | --- | --- | --- | --- | --- | --- |
| **PCB** | **S1** | **S2** | **S3** | **S4** | **S5** | **S6** | **S7** | **S8** | **S9** |
| 81 | 1.30E-13 | 1.30E-13 | 1.30E-13 | 1.30E-13 | 1.30E-13 | 1.30E-13 | 1.30E-13 | 1.30E-13 | 1.30E-13 |
| 77 | 3.40E-14 | 3.40E-14 | 3.40E-14 | 3.40E-14 | 3.40E-14 | 3.40E-14 | 3.40E-14 | 3.40E-14 | 3.40E-14 |
| 105 | 0.00E+00 | 1.20E-15 | 2.20E-15 | 1.40E-14 | 9.20E-16 | 2.00E-14 | 4.90E-15 | 1.90E-16 | 1.70E-15 |
| 114 | 2.60E-15 | 2.60E-15 | 2.60E-15 | 2.60E-15 | 2.60E-15 | 2.60E-15 | 2.60E-15 | 2.60E-15 | 2.60E-15 |
| 118 | 1.90E-15 | 1.90E-15 | 1.90E-15 | 1.90E-15 | 1.90E-15 | 1.90E-15 | 1.90E-15 | 1.90E-15 | 1.90E-15 |
| 123 | 1.10E-15 | 1.10E-15 | 1.10E-15 | 1.10E-15 | 1.10E-15 | 1.10E-15 | 1.10E-15 | 1.10E-15 | 1.10E-15 |
| 126 | 0.00E+00 | 6.60E-12 | 1.90E-11 | 3.10E-11 | 1.10E-11 | 1.00E-11 | 5.60E-12 | 7.50E-12 | 2.10E-11 |
| 156 | 3.00E-15 | 3.00E-15 | 3.00E-15 | 3.00E-15 | 3.00E-15 | 3.00E-15 | 3.00E-15 | 3.00E-15 | 3.00E-15 |
| 157 | 3.40E-15 | 3.40E-15 | 3.40E-15 | 3.40E-15 | 3.40E-15 | 3.40E-15 | 3.40E-15 | 3.40E-15 | 3.40E-15 |
| 167 | 2.80E-15 | 2.80E-15 | 2.80E-15 | 2.80E-15 | 2.80E-15 | 2.80E-15 | 2.80E-15 | 2.80E-15 | 2.80E-15 |
| 169 | 7.00E-12 | 7.00E-12 | 7.00E-12 | 7.00E-12 | 7.00E-12 | 7.00E-12 | 7.00E-12 | 7.00E-12 | 7.00E-12 |
| 189 | 1.30E-14 | 1.30E-14 | 1.30E-14 | 1.30E-14 | 1.30E-14 | 1.30E-14 | 1.30E-14 | 1.30E-14 | 1.30E-14 |
| Total | *7.20E-12* | *1.40E-11* | *2.70E-11* | *3.90E-11* | *1.80E-11* | *1.70E-11* | *1.30E-11* | *1.50E-11* | *2.80E-11* |

Table 4: Calculated Lifetime Cancer Risk via Ingestion for Non-dioxin-like Polychlorinated Biphenyls Across Study Sites

| **PCB** | **S1** | **S2** | **S3** | **S4** | **S5** | **S6** | **S7** | **S8** | **S9** |
| --- | --- | --- | --- | --- | --- | --- | --- | --- | --- |
| 28 | 2.00E-06 | 2.00E-07 | 2.10E-07 | 2.10E-07 | 1.80E-07 | 4.30E-07 | 4.20E-07 | 4.10E-08 | 4.60E-07 |
| 51 | 1.60E-06 | 2.10E-07 | 1.40E-07 | 1.40E-07 | 1.40E-07 | 1.60E-07 | 4.00E-07 | 1.10E-07 | 1.60E-07 |
| 101 | 7.20E-08 | 1.80E-08 | 1.30E-08 | 1.30E-08 | 2.80E-09 | 1.80E-08 | 7.00E-09 | 5.50E-09 | 3.80E-09 |
| 138 | 2.40E-08 | 5.30E-08 | 4.50E-09 | 1.40E-07 | 2.90E-08 | 8.70E-08 | 4.10E-08 | 2.70E-08 | 1.40E-08 |
| 153 | 6.00E-08 | 4.50E-08 | 1.00E-08 | 6.90E-08 | 4.10E-08 | 4.00E-08 | 2.40E-08 | 3.20E-08 | 3.60E-08 |
| 180 | 0.00E+00 | 0.00E+00 | 4.00E-09 | 2.20E-08 | 3.70E-08 | 4.00E-08 | 2.10E-08 | 1.30E-07 | 1.70E-07 |
| Total | *3.70E-06* | *5.20E-07* | *3.80E-07* | *5.90E-07* | *4.30E-07* | *7.70E-07* | *9.20E-07* | *3.50E-07* | *8.50E-07* |

| Table 5: Calculated Lifetime cancer Risk via Dermal Contact for Non-Dioxin-like Polychlorinated Biphenyls Across Study Sites | | | | | | | | | |
| --- | --- | --- | --- | --- | --- | --- | --- | --- | --- |
| **PCB** | **S1** | **S2** | **S3** | **S4** | **S5** | **S6** | **S7** | **S8** | **S9** |
| 28 | 1.00E-05 | 9.90E-07 | 1.10E-06 | 1.10E-06 | 9.10E-07 | 2.20E-06 | 2.10E-06 | 2.10E-07 | 2.30E-06 |
| 51 | 7.90E-06 | 1.10E-06 | 7.10E-07 | 7.10E-07 | 7.00E-07 | 7.90E-07 | 2.10E-06 | 5.40E-07 | 8.20E-07 |
| 101 | 3.60E-07 | 9.10E-08 | 6.50E-08 | 6.50E-08 | 1.40E-08 | 9.40E-08 | 3.60E-08 | 2.80E-08 | 1.90E-08 |
| 138 | 1.20E-07 | 2.70E-07 | 2.30E-08 | 7.10E-07 | 1.50E-07 | 4.40E-07 | 2.10E-07 | 1.40E-07 | 7.30E-08 |
| 153 | 3.10E-07 | 2.30E-07 | 5.20E-08 | 3.50E-07 | 2.10E-07 | 2.00E-07 | 1.20E-07 | 1.60E-07 | 1.80E-07 |
| 180 | 0.00E+00 | 0.00E+00 | 2.00E-08 | 1.10E-07 | 1.90E-07 | 2.00E-07 | 1.10E-07 | 6.80E-07 | 8.70E-07 |
| Total | *1.90E-05* | *2.60E-06* | *1.90E-06* | *3.00E-06* | *2.20E-06* | *3.90E-06* | *4.70E-06* | *1.80E-06* | *4.30E-06* |

Table 6: Calculated Lifetime Cancer Risk via Inhalation of Non-Dioxin-like Polychlorinated Biphenyls Across Study Sites

| **PCBs** | **S1** | **S2** | **S3** | **S4** | **S5** | **S6** | **S7** | **S8** | **S9** |
| --- | --- | --- | --- | --- | --- | --- | --- | --- | --- |
| 28 | 8.00E-11 | 8.00E-12 | 9.00E-12 | 9.00E-12 | 7.00E-12 | 2.00E-11 | 2.00E-11 | 2.00E-12 | 2.00E-11 |
| 51 | 7.00E-11 | 9.00E-12 | 6.00E-12 | 6.00E-12 | 6.00E-12 | 7.00E-12 | 2.00E-11 | 4.00E-12 | 7.00E-12 |
| 101 | 3.00E-12 | 7.00E-13 | 5.00E-13 | 5.00E-13 | 1.00E-13 | 8.00E-13 | 3.00E-13 | 2.00E-13 | 2.00E-13 |
| 138 | 1.00E-12 | 2.00E-12 | 2.00E-13 | 6.00E-12 | 1.00E-12 | 4.00E-12 | 2.00E-12 | 1.00E-12 | 6.00E-13 |
| 153 | 3.00E-12 | 2.00E-12 | 4.00E-13 | 3.00E-12 | 2.00E-12 | 2.00E-12 | 1.00E-12 | 1.00E-12 | 2.00E-12 |
| 180 | 0.00E+00 | 0.00E+00 | 2.00E-13 | 9.00E-13 | 2.00E-12 | 2.00E-12 | 9.00E-13 | 6.00E-12 | 7.00E-12 |
| Total | *2.00E-10* | *2.00E-11* | *2.00E-11* | *2.00E-11* | *2.00E-11* | *3.00E-11* | *4.00E-11* | *1.00E-11* | *4.00E-11* |
